# Digitalisation of forensic expert activity in Ukraine: Organisational and legal framework

**DOI:** 10.1016/j.fsisyn.2025.100578

**Published:** 2025-02-18

**Authors:** Nataliia Martynenko

**Affiliations:** Department of Law and Public Administration, Kyiv National University of Construction and Architecture, 31, Povitroflotsky Avenue, Kyiv, 03037, Ukraine

**Keywords:** Forensic expert activity, Digitalisation, Register of certified forensic experts, Profile of an expert organisation, Forensic expert e-cabinet

## Abstract

The article examines the process of digitalisation of forensic expert activity through the prism of the State's policy aimed at creating a technologically advanced society. Proposals are made: on registration and identification of forensic experts of state specialised institutions in the Unified Judiciary Information Telecommunication System; on improvement of the functions of the Register of Certified Forensic Experts regarding the profile of a legal entity carrying out forensic activities and the profile of an individual forensic expert. A number of legislative amendments related to the field of forensic science have been proposed to ensure a balanced development of the Register of Certified Forensic Experts.

The introduction of the Forensic Expert e-Diary will facilitate the maintenance of the Register of Certified Forensic Experts in terms of displaying the number of expert examinations conducted by each forensic expert and the level of their workload based on the number of examinations and their complexity, calculated in expert hours.

It is proved that the Register of Certified Forensic Experts should be maintained in such a way that the profiles of employed experts are displayed and made available via the profile of their expert organisation. It is concluded that in order to ensure independence, objectivity, efficiency, and speed of forensic examinations, it is advisable to introduce the following model of automated appointment of an expert to conduct a particular study: the system pre-selects several candidates, after which the court selects from the list an expert who can be involved in the work. The article also raises the issues of information security and data confidentiality in the organisation of forensic expert activity.

## Highlights

• Digitalisation of most of the work processes in forensic expert activity is in line with general digitalisation trends.
• Unification of the interagency approach to the classification of forensic examinations will facilitate a balanced development of the Register of Certified Forensic Experts.
• The profile of an expert organisation in the Register of Certified Forensic Experts should allow access to the profiles of the employed experts.
• The model of automated expert appointment will ensure independence, objectivity, efficiency, and speed of forensic examinations.
• The introduction of the Forensic Expert e-Diary will facilitate the maintenance of the Register of Certified Forensic Experts.
• Synchronisation of the Register of Certified Forensic Experts with the Unified Judiciary Information Telecommunication System (UJITS) will facilitate the exchange of information between these services.

## 1 Introduction

Since December 2021, the following subsystems (modules) of the Unified Judiciary Information Telecommunication System (UJITS) have been operating in Ukraine: e-Cabinet, e-Court, and videoconferencing subsystems [1,2].

The UJITS is a set of information and telecommunication subsystems (modules) that computerise the processes in courts, bodies and institutions of the justice system, including document management, computer-aided case distribution, exchange of documents between the court and litigants, recording of court proceedings and participation of litigants in court hearings via videoconference, preparation of operational and analytical reports, provision of information assistance to judges, as well as automation of processes that ensure financial, property, organisational, personnel, IT and other needs of the UJITS users [3].

Attorneys, notaries, public and private bailiffs, insolvency officers, forensic experts, public authorities and government bodies registered under the laws of Ukraine as legal entities, their territorial bodies, local governments, and other legal entities registered under the laws of Ukraine were required to register their electronic accounts in the UJITS or its separate subsystem (module) that provides for the exchange of documents [1].

The e-Cabinet is a user's personal account (web service or other user interface) in the UJITS subsystem (module), which provides a person who has passed electronic identification with access to information and services of the UJITS or its individual subsystems (modules), including the possibility of exchanging (sending and receiving) documents (including procedural documents, written and electronic evidence, etc.) between the court and parties to a trial, as well as between trial participants [4]. Electronic identification of a person is carried out using a qualified e-signature or other means of electronic identification that allow unambiguous identification.

The UJITS subsystem ‘e-Court’ provides for four types of User's e-Cabinets for: an individual, legal entity, lawyer, and judge.

The current procedural codes of Ukraine [[5], [6], [7], [8]] provide for the functioning of the UJITS and the mandatory registration of forensic experts’ electronic accounts in the UJITS.

There are certain problems with the registration and identification of forensic experts who are employees of state specialised institutions. Expert examinations are assigned to state specialised institutions, even if the court order specifies a particular expert. Employees of state specialised institutions do not have the right to conduct an examination without a written order from their supervisor, as this is a disciplinary offence under the law.

In the EU PRAVO-Justice Project Report “Summary of Discussions on Current Issues in the Field of Forensic Expertise”, as touching the introduction of an automated expert appointment model for conducting a particular study, it was noted that such innovations would help eliminate the human factor and ensure transparent selection of the most independent and objective expert, whose current workload makes it possible to conduct the study in a short time [9].

Therefore, the computerised distribution should not select an expert randomly, but take into account his or her current workload, specialisation and other criteria. It should be mentioned at the outset that the procedural legislation of Ukraine stipulates that the choice of an expert is the right of the litigants and the court. The automated allocation is not expressly stipulated in regulatory acts.

The ‘Engaging a Specialist’ function provided for in the user manual of the e-Cabinet of the UJITS is essentially analogous to the automated selection of an expert. However, the e-Court subsystem of the UJITS does not stipulate for a user's e-Cabinet for a forensic expert. There is a need to synchronise the Register of Certified Court Experts with the UJITS, so that these services can exchange information with each other.

## 2 Literature review

The advent of artificial intelligence (AI), software, algorithms and data programming, and their implementation in terms of expert systems, criminal investigations, predictability, etc. in the world of justice are an unstoppable reality [10]. All exchanges of information in the administration of justice should be carried out electronically [11]. To ensure an effective legal use of artificial intelligence in the judicial system, it is important to establish reliable safeguards and guarantee transparency [12].

The filing of documents is to be done digitally; therefore there is a need to develop a portal that allows parties to download documents after accessing it with personal credentials [13]. The digitisation of materials will increase the efficiency, accessibility and overall functionality of the justice system, since this form of facilitating access to files enhances the fair trial principle [14]. Cost-efficient storage of materials and better chances of safekeeping them for a sufficiently long time are also important [14]. The digitisation of case files is a prerequisite for introducing modern AI-based solutions into the justice system [14].

The Register of Certified Forensic Experts is not only a major selection tool, but also the one that sets benchmarks for standardising expert knowledge, and therefore provides a solid basis for building a well-established image of a forensic expert [15].

The use of a tamper-resistant method such as blockchain technology has several notable advantages and can be widely supported by forensic laboratories [16]. Blockchain technology can be used to efficiently collect evidence and maintain its integrity. It is this technology that can provide full transparency in the process of collecting, processing, and storing evidence [17]. Evidence can be collected at a crime scene only for once; thus mistakes, which result in evidence not being found, untested, lost, contaminated, invalidated, or incorrectly reported or passed on, can have catastrophic consequences for justice and the individuals or institutions involved [18].

Given the foregoing, the purpose of this article is to provide proposals for improving the functions of the Register of Certified Forensic Experts, introduction of computerised distribution of forensic examinations and the Forensic Expert e-Cabinet. For this purpose, it is supposed to consider the positive experience of forensic science digitalisation in some EU countries.

## 3 Methods

The systemic approach belongs to general methods and, as such, makes it possible to understand the integrity of the system in the unity of various state and legal phenomena that acquire new qualities, nonexistent when they are dissociated. The advantages of applying the systemic approach for completeness and comprehensiveness of the study allowed us to define both the problems of digitalising forensic expert activity in Ukraine and those of registering and identifying forensic experts of state specialised institutions in the Unified Judiciary Information Telecommunication System when organising forensic examinations, and on this basis, to provide proposals for improving the functions of the Register of Certified Forensic Experts, introduction of computerised distribution of forensic examinations and the Forensic Expert e-Cabinet, laying the basis for ensuring the quality of forensic expert activity.

The use of the structural and functional method made it possible to study in full the functional features of the Unified Judiciary Information Telecommunication System and the Register of Certified Forensic Experts and, on this basis, to consider the issues related to their synchronisation from different angles, to clarify the features and functional purpose of the Forensic Expert e-Cabinet, as well as its functional and systemic links with other modules of the Unified Judiciary Information Telecommunication System.

The method of comparative legal analysis provided for a comparison of the requirements for maintaining the Register of Certified Forensic Experts under the laws of Ukraine, and in some EU Member States.

The dialectical method, which is based on the complexity and comprehensiveness of the legal phenomena study, contributed to clarifying the problems of digitalisation of forensic expert activity and the introduction of a model for the computerised appointment of a forensic expert to conduct a particular study.

The scientific forecasting as one of the ways to achieve the purpose of the study is ensured by using the prognostic method. The application of the prognostic method in the context of the present study serves as a strategic vision of synchronising the Register of Certified Forensic Experts with the Unified Judiciary Information Telecommunication System and implementing a model for comuterised appointment of a forensic expert for a particular study. Modelling and forecasting methods made it possible to propose the content of the profiles of a legal entity carrying out forensic activities and of an individual forensic expert in the Register of Certified Forensic Experts.

## 4 Results

At present, the Register of Certified Forensic Experts, which is maintained by the Ministry of Justice of Ukraine [19], exists in the form of a list of experts, and its software is outdated. To introduce the interaction of the Register of Certified Forensic Experts with other state information systems, it is necessary to completely redesign the functionality of the existing software or create a new version of the said Register.

To this end, it is necessary to amend the current legislation to enable the interaction of the Register of Certified Forensic Experts with other state information systems and to regulate all aspects and features of such interaction between various exchange entities.

The key functionality resulting from the synchronisation of the Register of Certified Forensic Experts with the UJITS modules should include: processing requests for expert opinions, signing of expert opinions with a qualified electronic signature and attachment to court case files, etc.

It is important to confirm functionally that the document was signed by an expert who was authorised on the date of signing. Forensic experts should be checked by their qualified electronic signatures and status in the Register of Certified Forensic Experts. For a clear and unambiguous identification of the expert, it is necessary to have in the Register of Certified Forensic Experts at least the registration number of an individual taxpayer's account card or the unique identification number of a legal entity in the Unified State Register of Enterprises and Organisations of Ukraine, for which a qualified electronic signature has been issued.

It is worth noting that in France, every registered forensic expert is issued an expert card of the RGS 2∗/eIADS security level, which has an electronic data chip that allows verifying its authenticity and its holder. This smart card allows its holder: to access the OODRIVE/OPALEXE forensic dematerialisation platform; to sign electronically all types of documents, in particular, at the request of courts using the digital procedure (PPN); to sign electronically an expert report and send it using the PLEX tool [20]. In Austria, on the contrary, the Federal Ministry of Justice announced that from January 1, 2023, forensic experts are issued new identification cards that will no longer be equipped with a chip due to the cancellation of the certificate requirement [21].

The experience of cooperation in the field of forensic science between the Baltic States – Latvia, Lithuania and Estonia – shows the effectiveness of a unified classification of forensic examinations, which functions as a link between all the three interfaces of the Baltic Register of Forensic Examinations [22].

Given that forensic expertise is distinguished by the diversity of specialised knowledge used and the complexity of research procedures [23], it is important, before synchronising the Register of Certified Forensic Experts with the UJITS modules, to resolve legislatively the following issues:

- creation of an updated unified list of types of forensic examinations with their respective expert specialisations, which are used to award a forensic expert qualification, since without a unified interagency approach to classification it is difficult to avoid mistakes that can lead to unreasoned or illegal court decisions [23];
- taking an oath by a forensic expert after certification and obtaining a qualification, followed by entering this information into the Register of Certified Forensic Experts [24];
- protection of the rights of women and men to equal and non-discriminative obtaining of benefits – gender dimension should become an integral part of the process of developing, implementing, monitoring, and evaluating policies and activities in the field of forensic science [25];
- a proper building of the financial support for forensic activities [26,27].

In order to elaborate the issue of identification, it is advisable to use a forensic expert's personal data, which is contained in the introductory part of the opinion. However, it should be noted that this information differs in departmental instructions of organisations conducting forensic examinations. Therefore, there is a need to unify the requirements for information about a forensic expert, specified in the introductory part of the expert opinion: surname, name and patronymic; position, class of forensic expert that conducts forensic examination; education, educational qualification level, academic degree, academic title; expert specialisation; number and date of issuing the certificate of forensic expert qualification, the issuing organisation and its validity period; length of expert work, etc.

Let us again turn to positive foreign experience. In the Slovak Republic, the Ministry of Justice is responsible for maintaining the register of experts. The Ministry registers natural persons – experts; and legal entities – expert organisations and expert institutes. The profile of an expert organisation or institute contains the following information: name, location and identification number of the organisation; place of examination; surname, name, patronymic, academic degree, academic title, and position of the person responsible for the conduct of expertise, whose profile is available via the profile of the expert organisation or institute; areas of expert activity; dates of entry into and exclusion from the register; sanctions imposed in the last three years in connection with the professional activities; in case of prohibition of expert activity, the prohibition period; departments and branches at expert organisations, departments at expert institutes; information on temporary cessation of activities, including information on the legal grounds, start and end dates of temporary cessation of activities; information about the interruption of activities, including data on the start and end date of the interruption of activities; the term of the contract of liability insurance against damage that may arise in connection with the conduct of expertise and the name of the insurance company; contact data; employed experts whose profiles are available via the profile of the expert organisation or institute [28]. The profile of an individual expert contains the following information: surname, name, patronymic; academic title, academic degree; registration number; place of permanent residence; place of expertise; contact details (landline and mobile phone numbers, email address); dates of entry into and exclusion from the register; areas of expert activity; the validity of liability insurance against damage that may arise in connection with professional activities and the name of the insurance company; information on the temporary cessation of activities, including that on the legal grounds, start and end dates of the temporary cessation of activities; sanctions imposed during the last three years in connection with professional activities; in the case of a ban on the activity, the ban period; identification data of the employer or information on other practice in the field, if an expert's lack of practice is the basis for a decision to exclude him/her from the register of experts.

The Register of Experts of the Slovak Republic has the function ‘Show experts on the map’. The data entered in the list, except for the date of birth, permanent residence and legal basis for temporary suspension of activities, is publicly available; the Ministry of Justice ensures its publication on the website. Presently, one of the modules of the project “Development of Electronic Court Services” is being implemented – the Forensic Expert e-Diary, which will allow keeping current records of expert activities, sending expert reports to the central repository provided by the Ministry of Justice of the Slovak Republic, and simplifying the expertise accounting [29]. The expert is to keep a current diary of his/her activities in electronic form using soft- and hardware approved by the Ministry of Justice of the Slovak Republic and is responsible for the accuracy of the entered information [30].

Significantly, in France one of the conditions for the registration of individuals in the lists of forensic experts is non-carrying out any activities incompatible with the independence required to perform the duties of a forensic expert [31]. Among other prerequisites, in order to register a legal entity in the lists of forensic experts in France, it is also necessary to substantiate the experience in the chosen field of expertise; to prove the availability of appropriate technical means and qualified personnel, etc. The availability of sufficient equipment to provide an expert opinion in the relevant subject area is one of the conditions for inclusion in the list of forensic experts in Austria [32]. The conditions for inclusion in the list of forensic experts of the Czech Republic also include the availability of the necessary material and technical training and equipment to ensure a proper performance of expert work [33].

In Austria, an expert is selected by the court from a list of court-certified experts. The litigants have the option to refuse if there are reasonable doubts about the expert's impartiality [34]. In order to be included in the list of court experts in Austria, it is necessary to conclude a liability insurance contract with an insurer authorised to do business in Austria, and to maintain the insurance throughout the period of being on the list [32]. The list of court-approved experts in Austria is divided into groups of specialists, and within the groups of specialists – into specialised areas, taking into account the subject matter of their activity [32,35].

The List of Experts of the Czech Republic is a public-administrative information system in which experts, expert bureaus and expert institutes are registered, and for the maintenance of which the Ministry of Justice of the Czech Republic uses data from other registers and public-administrative information systems [33]. The List of Experts of the Czech Republic contains public information on when and for how long the expert activity was suspended and the grounds for that, as well as information on the offence committed in accordance with the Law on Experts, Expert Bureaus and Expert Institutes, and the administrative penalty imposed, if the resolution on the offence came into force less than 5 years ago [33]. An expert must notify the Ministry of Justice of the Czech Republic of any changes in the data entered in the list of experts within 10 working days from the date of such change [33]. In Slovenia, forensic experts must notify in writing within three days of the following changes: the postal address at which they are available; permanent or temporary residence address; contact telephone numbers or e-mail addresses; and information about the place of work [36]. In case of failure to notify of the above, the expert is subject to disciplinary action.

The Ministry of Justice, Administration and Digital Transformation of the Republic of Croatia maintains lists of appointed permanent forensic experts and legal entities authorised to carry out expert work and publishes them on the electronic notice boards of the courts [37,38]. In Slovenia, data on a person is archived upon removal from the directory of forensic experts. The Ministry of Justice of the Republic of Slovenia ensures the permanent storage of archived data in accordance with the legislation governing the protection of personal data [36]. The Austrian Federal Government is liable for any damage caused by the use of automated data processing due to errors in the maintenance of the list of forensic experts [32].

Ukraine is about to raise the issue of creating an electronic archive of forensic expert opinions in the UJITS. It is advisable to store them across court cases recorded in the judicial system within the automated court document management system and its central database. There will be a need to integrate the electronic document management systems of state specialised institutions into the UJITS in order to store all documentation related to the work of a forensic expert in electronic form.

The use of blockchain technology to maintain the integrity of evidence in the field of forensic science and improve the reliability of the chain of custody in court cases will help reduce human error, which will guarantee the speed, reliability and security of the justice system [17]. To implement this technology, a multidisciplinary approach involving computer scientists, forensic experts, lawyers, and policy makers is recommended [39]. The creation of specialised task forces or working groups will facilitate the development of standardised organisations, best practices and training programmes adapted to the specific needs of different jurisdictions and forensic organisations [39].

Among the above-mentioned areas of digitalisation of forensic expert activity, special attention should be paid to information security in the work of state specialised institutions, expert institutions of municipal ownership, as well as forensic experts who are not employees of these institutions. It is necessary to resolve the issue of their obtaining certificates of the ‘Integrated Information Security System’ – a system encompassing an interconnected set of organisational and engineering measures, tools and methods to ensure the integrity of data in information systems and the implementation of information security policies to prevent unauthorised interference with IT systems and information leakage, and to ensure information security in the workplace.

## 5 Suggestions for Ukraine

According to the results of the study, and with account of foreign experience, the profile of a legal entity carrying out forensic expertise in the Register of Certified Forensic Experts should include: the name, location, and identification code of the legal entity; place of expertise; surname, name, patronymic, academic degrees, academic titles, posts of managers whose profiles are available via the organisation's profile; areas of expert activity; dates of entry into and exclusion from the Register of Certified Forensic Experts; sanctions imposed in the last three years in connection with the professional activities; in case of prohibition of expert activity – the prohibition period; separate subdivisions (branches); information on temporary cessation of activity with reference to the legal grounds, the dates of commencement and termination of temporary cessation of activity; information on interruption of activity, including data on the dates of commencement and termination of interruption of activity; the term of the contract of liability insurance against damage that may arise in connection with the performance of expert work and the name of the insurance company; contact details; availability of appropriate technical means and equipment required to provide an expert opinion; forensic experts employed whose profiles are available via the organisation's profile.

In order to introduce a computerised selection of experts for courts and customers of expertise through the synchronisation of the Register of Certified Forensic Experts and the USITS, it is necessary to conduct an additional analysis of the Register of Certified Forensic Experts, prepare a detailed analytical description of the functionality so that the system produces a list of operating individuals – forensic experts, indicating: types of expertise that the expert can perform; surname, name and patronymic; date of birth; taxpayer registration number; place of work (business address); contact details (landline and mobile phone numbers, email address); date of entry into and exclusion from the Register of Certified Forensic Experts; availability of liability insurance against damage that may arise in connection with professional activities and the name of the insurance company; number and date of issue of the certificate of a forensic expert qualification, the issuing institution and validity period; expert specialisation; the class of the forensic expert entrusted with conducting forensic examination; length of expert work; education, educational qualification level; academic degree and academic title; identification data of the employer, and a position held – for employees of state specialised institutions; disciplinary sanctions imposed within the last five years, if any; information on the temporary cessation of activities, indicating the legal grounds, start and end dates of the temporary cessation of activities; the number of forensic examinations conducted; the level of workload based on the number of examinations and their complexity, calculated in expert hours; in case of a ban on activities, the period during which the ban is in force.

For a substantiated development of the Register of Certified Forensic Experts in Ukraine, a number of legislative forensics-related changes should be introduced:

- unification of the interagency approach to the classification of forensic examinations;
- taking of an oath by a forensic expert;
- unification of the requirements of departmental instructions as to information about a forensic expert, which is specified in the introductory part of the expert opinion;
- protecting the rights of women and men to equality and non-discrimination in order to obtain equivalent benefits;
- insurance of a forensic expert against the potential professional liability;
- nonperformance by forensic experts of any activity incompatible with the independence principle in carrying out professional duties;
- notification of changes in the data entered in the Register of Certified Forensic Experts within the established period, and liability for failure to fulfill this obligation in a timely manner.

## 6 Conclusions

The introduction of an automated model for appointment of a forensic expert to conduct a particular study will help eliminate the human factor and ensure the transparent selection of the most independent and objective expert, whose current workload makes it possible to conduct the study in a short time. However, the research has not revealed a model among those used in the EU member states where the court would be deprived of the right to choose a forensic expert and this right would be transferred to automated systems. Yet, the option of partial computerised distribution of cases between experts is still possible. In order to ensure the independence, objectivity, efficiency and expedience of forensic examinations, it would be advisable to introduce a model of computer-aided appointment of an expert to conduct a particular study, so that the system pre-selects several candidates, and then the court selects from the list an expert who is suitable for that work.

The Register of Certified Forensic Experts should register both individuals – forensic experts, and legal entities – state specialised institutions: forensic research institutions, forensic medical and forensic psychiatric institutions, and expert services. It is expedient to maintain the Register of Certified Forensic Experts in such a way that the profiles of experts are displayed and accessible via the profile of their expert organisation.

The idea of introducing the Forensic Expert e-Diary seems quite useful, since this will allow for the current accounting of expert activities using approved soft- and hardware. The Forensic Expert e-Diary will facilitate the maintenance of the Register of Certified Forensic Experts in terms of registering the number of expert examinations conducted by each forensic expert; the level of workload based on the number of examinations and their complexity, calculated in expert hours, etc.

It is advisable to consider the possibility for judges to place in the Register of Certified Forensic Experts information about unreasonable refusal to conduct an expert examination, poor quality of expertise, as well as about significant violations of the deadlines for performance of forensic examinations.

It is necessary to digitalise most of the work processes in forensic expert activity, namely: to create an electronic form of an expert opinion and send its electronic copy to all participants in the case; to store digitalized documents in the electronic systems of the relevant expert institution, etc., which is fully in line with the general digitalisation trends.

The digitalisation of forensic activities necessitates the development of a security policy and establishing the main provisions of information security management. The lack of preventive measures in the field of information security can be a significant problem for forensic institutions.

## Consent for publication

The author gives full consent for the publication of the article on approval.

## Ethics approval and consent to participate

The given manuscript is a review article. Therefore, there is no requirement for ethical approval or participation consent. This article does not contain any studies with human participants or animals performed by the authors.

## Availability of data and material

All required data pertaining to the manuscript has been already provided. There is no supplemental data.

## Data availability

There are no data and material other than the manuscript.

## Compliance with ethical standards

This article does not contain any studies with human participants or animals performed by the authors. Ethics declaration: not applicable.

## Funding

This research received no specific grant from any funding agency in the public, commercial, or not-for-profit sectors. This research received no external funding. This study was not funded.

## Declaration of competing interest

There is no conflict of interest.

## References

[1] Verkhovna rada Ukrainy (2017). On amendments to the economic procedural code of Ukraine, the civil procedural code of Ukraine, the code of administrative procedure of Ukraine and other legislative acts: law of Ukraine dated october 3 № 2147-VIII. https://zakon.rada.gov.ua/laws/show/2147%D0%B0-19#Text.

[2] Judicial power of Ukraine (2021). Announcement on the creation and maintenance of the operation of three subsystems (modules) of EUITS. https://court.gov.ua/press/news/1173657.

[3] Supreme Council of Justice (2021). On the approval of the regulation on the procedure for the functioning of individual subsystems of the unified judicial information and telecommunication system: decision of august 17 № 1845/0/15-21. https://zakon.rada.gov.ua/rada/show/en/v1845910-21?lang=uk#Text.

[4] Verkhovna rada Ukrainy (2023). On amendments to some legislative acts of Ukraine regarding mandatory registration and use of electronic cabinets in the Unified Judicial Information and Telecommunication System or its separate subsystem (module) that ensures the exchange of documents: law of Ukraine dated June 29 № 3200-IX. https://zakon.rada.gov.ua/laws/show/3200-20#Text.

[5] Verkhovna rada Ukrainy (1991). Economic procedural code of Ukraine: law of Ukraine dated november 6 № 1798-XII. https://zakon.rada.gov.ua/laws/show/1798-12#n1777.

[6] Verkhovna rada Ukrainy (2004). Civil procedure code of Ukraine: law of Ukraine dated march 18 № 1618-IV. https://zakon.rada.gov.ua/laws/show/1618-15#n6136.

[7] Verkhovna rada Ukrainy (2005). Administrative judicial code of Ukraine: law of Ukraine dated july 6 № 2747-IV. https://zakon.rada.gov.ua/laws/show/2747-15#n9666.

[8] Verkhovna rada Ukrainy (2012). Criminal procedure code of Ukraine: code of Ukraine dated april 13 № 4651-VI. https://zakon.rada.gov.ua/laws/show/4651-17#Text.

[9] (2023). Generalization Based on the Results of Discussions of Current Issues in the Field of Forensic Examination.

[10] Barona Vilar S. (2019). Inteligencia Artificial o la algoritmización de la vida y de la Justicia: ¿solución o problema?. Revista boliviana de derecho.

[11] Planchadell-Gargallo A. (2024). Breves apuntes sobre digitalización del proceso penal español a la luz de la reciente reforma. Revista Brasileira De Direito Processual Penal.

[12] Xavier P.R.S. (2024). Smartjusticia, proceso y prueba: especial referencia a su uso en los tribunales de apelación. Revista Brasileira De Direito Processual Penal.

[13] Vicoli D. (2024). Editorial do dossiê “Digitalização e justiça penal: aspectos procedimentais” – Uma introdução geral. Revista Brasileira De Direito Processual Penal.

[14] Sakowicz A., Zieliński S. (2024). Em direção a um sistema de justiça criminal digitalizado: exemplo da Polônia. Revista Brasileira De Direito Processual Penal.

[15] Renard B. (2017). La figure instituée de l’expert judiciaire. Enjeux autour de l’instauration d’un registre national des experts judiciaires. Pyramides.

[16] McAndrew W.P., Speaker P.J., Houck M.M. (2023). Interpol review of forensic management, 2019-2022. Forensic Sci. Int..

[17] Patil H., Kohli R.K., Puri S. (2024). Potential applicability of blockchain technology in the maintenance of chain of custody in forensic casework. Egypt J Forensic Sci.

[18] Heavey A.L., Houck M.M. (2024). Rethinking scientific communication in courts: a question of credibility. Forensic science international. Synergy.

[19] Verkhovna rada Ukrainy (1994). On forensic examination: law of Ukraine dated February 25 № 4038-XII. https://zakon.rada.gov.ua/laws/show/4038-12#Text.

[20] Conseil National des Compagnies d'Experts de Justice (2024). Carte d'expert. Dématérialisation. Accueil. Conseil National des Compagnies d'Experts de Justice. https://www.cncej.org/carte-exxpert.

[21] Guggenbichler J. (2023). Hauptverband der allgemein beeideten und gerichtlich zertifizierten sachverständigen österreichs.

[22] Sillon N. (2019). https://experts-institute.eu/en/expertise-law-and-jurisprudence/the-baltic-register-of-forensic-science-experts/.

[23] Martynenko N., Domin D. (2023). Expert Assessments in Decision Making: Risks and Safety.

[24] Martynenko N. (2024). Areas of modernization of the system of training and certification of forensic experts in Ukraine based on the adaptation of foreign experience. Forensic Sciences Research.

[25] Martynenko N. (2024). The mechanism for ensuring gender equality in the field of forensic expertise: a balanced gender policy. Balkan Social Science Review.

[26] Martynenko N. (2024). Directions for improving the financial support of forensic expert forensic activity in Ukraine with account of positive foreign experience. Forensic Sci. Int.: Synergy.

[27] Martynenko N. (June 2025). Methodological support for forensic science in the USA and Ukraine: a comparative study. Forensic Sci. Int.: Synergy.

[28] Ministerstvo spravodlivosti Slovenskej republiky (2024). Znalci. Registre. https://www.justice.gov.sk/registre/znalci/?stav_string=zapis&rozhodnyDatum=15.08.2024&pageNum=1&size=10&sortProperty=meno_sort&sortDirection=ASC.

[29] Ministerstvo spravodlivosti Slovenskej republiky (2024). Elektronický denník znalca, tlmočníka a prekladateľa. Znalci. Služby. https://www.justice.gov.sk/sluzby/znalci/elektronicky-dennik-znalca-tlmocnika-a-prekladatela.

[30] Národná rada Slovenskej republiky (2004). ZÁKON № 382 z 26. mája o znalcoch, tlmočníkoch a prekladateľoch a o zmene a doplnení niektorých zákonov. https://www.slov-lex.sk/pravne-predpisy/SK/ZZ/2004/382/.

[31] Droit national en vigueur (2004). Textes consolidés. Décret n°2004-1463 du 23 décembre 2004 relatif aux experts judiciaires. https://www.legifrance.gouv.fr/loda/id/LEGISCTA000006083039.

[32] Rechtsinformationssystem des Bundes (1975). Bundesgesetz über die allgemein beeideten und gerichtlich zertifizierten Sachverständigen und Dolmetscher (Sachverständigen- und Dolmetschergesetz – SDG) StF: BGBl. https://www.ris.bka.gv.at/GeltendeFassung.wxe?Abfrage=Bundesnormen&Gesetzesnummer=10002338.

[33] Parlament České republiky (2019). Zákon o znalcích, znaleckých kancelářích a znaleckých ústavech: 09.10.2019 № 254/2019. https://www.zakonyprolidi.cz/cs/2019-254.

[34] ID Austria (2024). Beteiligte im Zivilprozess. Zivilrecht. Gerichtsorganisation der Justiz. Gesetze und Recht. Themen.

[35] JustizOnline – die digitalen Informations- und Serviceangebote der österreichischen Justiz (2024). Allgemein beeidete und gerichtlich zertifizierte Sachverständige. Sachverständige. Sachverständige & Dolmetscher:innen.

[36] Državni zbor R.S. (2018). Zakon o sodnih izvedencih, sodnih cenilcih in sodnih tolmačih (ZSICT): 22. 3. 2018 № 2530-VII. https://pisrs.si/pregledPredpisa?id=ZAKO7726.

[37] Ministarstvo pravosuđa i uprave Republike Hrvatske Istaknute teme. Popis stalnih sudskih vještaka i stalnih sudskih procjenitelja. https://mpudt.gov.hr/UserDocsImages/26670.

[38] Stalni sudski vještaci Ministarstva pravosuđa, uprave i digitalne transformacije. https://mpudt.gov.hr/istaknute-teme/stalni-sudski-vjestaci/26662.

[39] Soni N. (2024). Letter to the editor: “Potential applicability of blockchain technology in the maintenance of chain of custody in forensic casework”. Egypt J Forensic Sci.

